# Supplementary material for: Smoking trajectories and risk of stroke until age of 50 years – The Northern Finland Birth Cohort 1966
Source: PLoS One. 2019 Dec 17;14(12):e0225909. doi: 10.1371/journal.pone.0225909 (PMC6917292; doi:10.1371/journal.pone.0225909)
Supplement: S1 Table — (DOCX) [file pone.0225909.s001.docx]

**S1 Table. Variables used in the multiple imputation procedure.**

|  | **% of missing data** | | **Imputed** | **Used only as predictor in MI** |
| --- | --- | --- | --- | --- |
| **Predictors in Cox regression model** | | |  |  |
| Smoking status at age 14 | | 9.6 | x |  |
| Smoking status at age 31 | | 28.5 | x |  |
| Smoking status at age 46 | | 43.5 | x |  |
| Starting age of smoking | | 51.4 | x |  |
| Ending age of smoking | | 53.5 | x |  |
| Pack-years by age 31 | | 43.2 | x |  |
| Pack-years by age 46 | | 67.8 | x |  |
| **Covariates** | |  |  |  |
| Sex | | 0 |  | x |
| Alcohol consumption at age 46 | | 44.5 | x |  |
| BMI at age 46 | | 41.0 | x |  |
| Hypertension at age 46 | | 40.5 | x |  |
| Diabetes at age 46 | | 40.4 | x |  |
| Hypercholesterolemia at age 46 | | 40.8 | x |  |
| Physical activity at age 46 | | 46.9 | x |  |
| Family history of stroke | | 41.8 | x |  |
| Educational level by age 46 | | 22.0 | x |  |
| **Outcome variables** | |  |  |  |
| Diagnosis of IS | | 0 |  | x |
| Diagnosis of TIA | | 0 |  | x |
| Diagnosis of SAH | | 0 |  | x |
| Diagnosis of ICH | | 0 |  | x |
| Other stroke diagnosis | | 0 |  | x |
| Age of stroke onset | | 97.2 | x |  |
| **Other variables** | |  |  |  |
| Mother’s smoking during pregnancy | | <5.0 | x |  |
| Father’s smoking during adolescence | | 15.9 | x |  |
| Passive smoking at age 31 | | 28.2 | x |  |
| Occupational status of mother | | <5.0 | x |  |
| Occupational status of father | | 5.0 | x |  |
| Alcohol use at age 14 | | 9.6 | x |  |
| Alcohol consumption at age 31 | | 30.2 | x |  |
| BMI at age 14 | | 15.7 | x |  |
| BMI at age 31 | | 28.2 | x |  |
| Hypertension at age 31 | | 26.0 | x |  |
| Diabetes at age 31 | | 27.6 | x |  |
| Hypercholesterolemia at age 31 | | 27.3 | x |  |
| Physical activity at age 31 | | 30.0 | x |  |
| Use of other drugs at age 14 | |  |  | x |
| Problems with alcohol at age 31 | |  |  | x |
| Problems with alcohol at age 46 | |  |  | x |
| Problems with other intoxicants at age 31 | |  |  | x |
| Problems with other intoxicants at age 46 | |  |  | x |
| Circumference of pelvis at age 31 | |  |  | x |
| Circumference of waist at age 31 | |  |  | x |
| Circumference of pelvis at age 46 | |  |  | x |
| Circumference of waist at age 46 | |  |  | x |
| Total cholesterol level at age 31 | |  |  | x |
| Total cholesterol level at age 46 | |  |  | x |
| Blood insulin level at age 31 | |  |  | x |
| Blood insulin level at age 46 | |  |  | x |
| Diagnosis of any mental disorder | |  |  | x |
| Self-reported medication for cardiovascular disorders at age 31 | |  |  | x |
