# Supplementary material for: Smoking trajectories and risk of stroke until age of 50 years – The Northern Finland Birth Cohort 1966
Source: PLoS One. 2019 Dec 17;14(12):e0225909. doi: 10.1371/journal.pone.0225909 (PMC6917292; doi:10.1371/journal.pone.0225909)
Supplement: Attachment — Submitted filename: Line by line comments_160819.docx [file pone.0225909.s002.docx]

**Smoking trajectories and risk of stroke until age of 50 years – the Northern Finland Birth Cohort 1966 (PONE-D-19-20624)**

**Title page:**

Line 4: It says that the author Ina Rissanen is part of the Department of Neurology and Neurosurgery and Medical Research Center Oulu (lines 7-9). However, in the submission data it is stated that Ina Rissanen is from the Universitair Medisch Centrum Utrecht.

**Abstract**:

Line 24: I would suggest to use a plural with “trajectory of smoking exposure” (so trajectories of…). After all, your aim is to study how the distinct smoking trajectories are related to stroke risk – not to identify a single growth trajectory that assumes the individuals to come from a single population and can be described and approximated with one trajectory.

Line 24: “Life course” is spelled without the hyphen. Correct throughout the MS.

Line 25: “Aimed” should be written in small letters.

Line 25: The sentence would be clearer if you would use the term relationship / association here as well as you did in the background description.

Lines 24–25: I would like to see you use the same term of the trajectories throughout the MS (either smoking exposure trajectories / trajectories of smoking exposure).

Line 28: Comma after the 1966 or rephrasing the sentence. It is difficult to read when two numbers are following each other.

Lines 29–30: You also gathered data on smoking status at three age points. Mention that as well (or simply combine the smoking variables by stating that smoking behavior was assessed at ages 14, 31 and 46 years).

Lines 28–31: There is nothing about the statistical methods in the abstract. If the word count do not limit you too much, you might add a phrase on that.

Line 30: Do not use abbreviations (ICD) in abstract.

Line 33: Comma before the 352 or rephrasing the sentence.

Line 34: Put the statistical analyses in Methods, not in Results.

Lines 29–30, 39: It is not clear in the abstract nor yet in the methods of the MS what you mean by cumulative smoking and what are smoked pack-years. Please clarify the terms you use.

**Introduction**:

Line 43: According to the reference number 1, I believe that stroke is the second largest cause of death, but the **third** for disability. Please check the reference again and be precise.

Line 45: You use the terms “young people”, “younger age” and “young age” in your MS. Do you always refer to people under 50 years (like in line 45) with these terms? Generally, it feels strange to use the term “young” when referring also to middle-aged people – even though I understand that the term “young” is used here since this is a young age to have a stroke. Nonetheless, please define somewhere (in the introduction or in the methods) what exact age you mean by “young” in the whole MS, use the same term throughout the MS, or consider using some other term (e.g., people under 50 years of age / young adults) to avoid confusion.

Lines 45–48: Divide to two sentences from “however” onward. I suggest to do the same with many other long sentences (e.g., in lines 49–51, 55–58, 58–60). Check this throughout the text. Just to make the text more fluent, use also some other term than “however” to avoid repetition (e.g., nonetheless).

Line 53: Again, I would wish precision with terms. What does “later in life” and “younger age” mean here? Could you give specific age?

Lines 58–59: The references to these “Most previous studies” are missing. Additionally, there are previous studies that have already identified diverse smoking trajectories, e.g.:

- Artaud et al. Trajectories of unhealthy behaviors in midlife and risk of disability at older ages in the Whitehall II Cohort Study. The Journals of Gerontology 2016, 71 (11), 1500-1506.
- Brook et al. Developmental trajectories of cigarette smoking from adolescence to the early thirties: personality and behavioral risk factors. Nicotine & Tobacco Research 2008, 10(8), 1283–1291.
- Chassin et al. Multiple trajectories of cigarette smoking and the intergenerational transmission of smoking: a multigenerational, longitudinal study of a Midwestern community sample. Health Psychology 2008, 27(6), 819-828.
- Salin et al. Smoking and physical activity trajectories from childhood to midlife. Int. J. Environ. Res. Public Health 2019, 16(6), 974.

Please acknowledge the use of trajectory modelling in smoking studies. I suggest for you to use these findings either in the introduction or discuss the similarities and differences of the findings of other trajectory studies and compare them to your own findings in the Discussion.

Line 60: This is a bit difficult phrase to understand: “…they have different patterns of future smoking habits”. Could it just be “…they have diverse / distinct /differing smoking patterns”?

Lines 62–64: The reasoning for this study in lines 63-64 is good. However, references are missing again. What are the “most previous studies”? And how short is the “short follow-up”? Be more specific, please.

Line 66–67: Why not simply “…the effect of smoking on stroke risk **depended** on…”?

Line 69: Are the words “…effect of smoking **to stroke risk…**” missing here? Additionally, do you mean “during adolescence” or would “starting from adolescence” be more accurate in this context?

Line 74: Define “cumulative smoking” here or in the Methods (preferably the first time you mention it).

Line 77: Would it be possible to use the exact age here (not the term “childhood”)?

**Methods**:

Lines 82–105: I suggest you to give a subheading (3.1) for these paragraphs as well (e.g., Design and population of the study).

Line 88: If accessible, give information of reasons why participants declined use of their data, and what were the reasons for non-participation later in the study (loss to follow-up).

Line 92: Please state the average follow-up time per participant in the current study.

Line 92: Add L to “hea**l**th”.

Lines 98–102: If possible, divide to two sentences (long and difficult to understand).

Line 102: Would it be possible to mention the registers’ names here since you talk about them here for the first time?

Lines 107–114: It is not stated clearly whether the smoking variable is binary at all three age points (14, 31, and 46 years). It seems it’s binary at the age of 14 and has three categories at the age of 31 and 46. However, on the line 174, you state that the smoking variables are binary.

Line 119: I suggest you to define in here the terms smoked pack years and cumulative smoking.

Line 123: This is the first time you use TIA. Please write the whole term open before using abbreviations.

Lines 127–128: The reference is missing.

Line 138: State in the 3.3 section the age points of the covariates.

Lines 139–140: In the text you use terms low, moderate and high education and in the table 2 terms basic, secondary and tertiary. Be consistent throughout the text. Additionally, define what these education levels mean.

Lines 140–141: The amount of physical activity seems rather high in table 2. Please define here more accurately how physical activity is assessed (e.g., does it include intensity, frequency, organized and unorganized activity, commuting, household work, leisure-time physical activity…).

Line 160: You use the term outcome on this line but it has not been defined in the text yet. It would be good to actually state somewhere that smoking is the exposure and stroke is the outcome.

Line 164: It is not necessary to write an abbreviation (here MAR) if you will not use it again in the text. Check this with all the abbreviations used.

Line 169: It is good that you made sensitivity analysis. However, the supplement 2 is not attached to the submission. Please add it.

Line 173: Just to avoid confusion, I suggest not to use the term “cluster” with trajectory modelling since cluster analysis is a different type of statistical method. I suggest the use of trajectory, group, subgroup or class.

Line 175: (This is a side note for possible future trajectory modelling.) Usually binary variables are not used in trajectory modelling in order not to lose valuable information. Thus, if you would have a variable of smoking with wider scale (e.g., how many cigarettes does the participant smoke in a day), it might be more suitable for trajectory modelling. Nonetheless, you have solved the problem by using logit-based model which is acceptable.

Line 175: It is recommended that also the model with one trajectory class is tested and reported (see Schoot et al. The GRoLTS Checklist: Guidelines for Reporting on Latent Trajectory Studies. Structural Equation Modeling: A Multidisciplinary Journal 2017, 24: 451–467.). Thus, read carefully this paper and then improve your reporting of the statistics and results concerning smoking trajectories. Try to report all the points mentioned in the Table 1 (Final List of Items of the Guidelines for Reporting on Latent Trajectory Studies) in the Schoot’s et al. article. Additionally, a table with the information from all the tested trajectory models (models 1–6) is needed in the Results. An example of this table (Table 2: Example Table With Hypothetical Results) is found in the above-mentioned article. It is important that the reader has the possibility to see the information criterion values, entropy values, average posterior probabilities and sample sizes per class of the different models in order to evaluate what is the importance given to the clinical significance of the modelling and what for the fit indices when choosing the final model.

Line 186: Since the class membership might change slightly when adding outcomes, predictors, covariates, etc. to the model, it would be more advisable to do all the analyses within the same modelling meaning the identification of trajectories and, in your case, the outcome (stroke). This would be possible by using Mplus. Unfortunately, I am not that familiar with SAS so I cannot say if you could do all these analyses by using SAS (and not SPSS). However, I think your solution is acceptable – especially if the posterior probabilities of the classes are high enough in all of the five classes and you will report them in the Results.

Line 189: Add Supplement **1**?

Line 189: Change to “starting and **ending** age”?

Line 190-191: Why adjusted only for smoked pack-years?

**Results**:

Line 196: Define person years.

Line 197: Add “smoking” and shorten the end of the sentence to “…five different smoking patterns were detected”.

Line 202: Could you consider deleting the part “by themselves at least”? Gives an impression that these persons have only smoked alone.

Line 203: I would change “tried smoking” to “smoked”. This gives an impression that one should really try to be a smoker.

Line 203: How much is “very short”? Less than a month? Less than a year? Be more specific.

Line 204: “Ever smoker” sounds odd. Could it be “…who had smoked regularly”? Or what you are looking for here?

Line 214: The abbreviation TIA has been used previously in line 123. There is no need to write it open here (but you need to write it in line 123).

Lines 215–217: Would it be possible to show whether the ages (42.5 and 37.3) differ significantly from one another? You discuss a bit of the matter in the Discussion which is informative. However, could you be more profound in Discussion (e.g., what does previous literature say about why the onset of ischemic stroke comes later than onset of haemorrhagic stroke? This matter has apparently been studied in reference number 5. Please discuss whether / how your findings support these previous findings.)

Line 221: This heading could be misunderstood. It could mean that you talk about smoking risk and stroke risk. I believe you want to point out how smoking is a risk factor for stroke. Please rephrase.

Line 224: When compared to who?

Line 226: “…a**n** increased risk…”

Line 231: Define cumulative smoking earlier in the MS.

Line 241: The HR of haemorrhagic stroke are rather different than in the original analyses. Additionally, the CI is rather high - probably due to low number of strokes. It’s good that you talk about this limitation in the Discussion. However, please also discuss about the differences found between results of sensitivity analyses and original results in the Discussion.

Line 245: Mark reference to the supplement material 2 (I assume you will add it to the MS)?

**Discussion**:

Lines 249–322: The first paragraph concerning the main findings is concise. However, usually the strengths and the limitations of the study are discussed in the end of Discussion. Thus, the second and third (and maybe even the fourth) paragraphs are in a bit unconventional place. Also, the Discussion between these results and previous findings could be more profound. Would it be possible, for example, to discuss more about the meaning of this study when compared to previous findings in this area (e.g., smoking trajectories from childhood to adulthood, associations of smoking and stroke in other high-income countries, etc.).

Line 249: “…a**n** increased…”

Line 250: “…early quit**ters of smoking**…”

Line 251: “… stroke **when compared to non-smokers**.”

Line 264: This might be a good place to discuss the results from the sensitivity analyses.

Line 268: Reference is missing.

Lines 272–279: Some repetition within the paragraph. Please, truncate.

Lines 275 and 277–278: Do you have knowledge about the magnitude and direction of the potential bias?

Lines 278–279: Use same tense within the sentence (now present and imperfect).

Lines 282–283: Please, do not write in plural (previous studies) if you only have one study as reference. Nonetheless, this is an interesting point.

Lines 283–285: The ending comes too late and the phrase is difficult to understand on the first read. Could it be: “By contrast, a study examining young women found that, not the duration of smoking, but the dose of daily smoking…”

Lines 285–287: Divide into two sentences. Period after “pack years”. A possibility to start the next sentence would be: “Instead, the smoking trajectories…”.

Line 291: Reference is missing for the part “continuous smoking is related to higher cumulative **smoking** exposure”.

Lines 293–294: Doesn’t the Table 2 show that, indeed, the highest smoking pack years are among the continuous smokers?

Lines 294–295: References are missing concerning the stroke being a rare phenomenon among young adults and concerning the strokes in Finland. Also, delete the preposition “in” in line 295.

Lines 289–305: This paragraph also discusses a bit about limitations. Could it be placed close to those paragraphs concerning limitations?

Lines 298–300: Indeed, if most of the participants started smoking at 16 years of age, the number of participants who started smoking at other ages might be that small that it explains why no associations were found. By contrast, I am not sure if the starting age at 16 explains why differences could not be found between the other trajectories and stroke. If trajectory modelling identified the five distinct subgroups, they, in fact, should be distinct – if not by starting age of smoking then for some other reason. Of course, if there are many small trajectory classes, they might not have enough statistical power to show statistical differences. Nonetheless, the 16 years of age might not be the explanation.

Lines 301–305: Here the discussion between the current and previous findings is interesting. However, in the reference number 32 the researchers have not identified smoking trajectories. They use linear regression in their analyses. Naturally, you may refer to this reference, but use accurate terminology.

Line 307: Delete the first “as”. Also, sometimes you write “pack-years” and sometimes “pack years”. Choose one or the other and use it throughout the text.

Lines 308–311: If this is the case, why not to identify the smoking trajectories by using the cumulative smoking variable? If not for this study, maybe for the next one to compare which works better.

Line 312: “Childhood smoking” was slightly unexpected term since not many children smoke. Please check the reference number 34: do they really have this result concerning childhood smoking or do they mean the duration of smoking from childhood onward?

Lines 314–316: Again, do not write in plural (recent studies) if you only have one study as reference. Nonetheless, this is also an interesting point. Thus, what recent studies (line 314)? What previous studies (line 315)? Also, this would be a good place to mention one of the strengths of your study which actually followed the changes in smoking habits from childhood to adulthood. This is a strength of trajectory modelling in longitudinal studies. Other strengths of trajectory modelling can be found, for example, in the following reference:

- Nagin DS. Group-based modeling of development. Cambridge, Mass.: Harvard University Press; 2005.

Lines 319–321: Reference is missing.

Lines 325–328: I ask you to rethink your conclusions. First, indeed, your findings show that cumulative exposure to smoking is a risk factor for stroke. The next part is a bit inconsistent, though: you conclude that the trajectories were not associated with stroke risk even though you have reported in Results and in Discussion that also continuing smoking trajectory is associated with stroke risk. Be consistent with reporting. Also, consider whether you can conclude that starting age of smoking is not crucial for stroke risk if the reason for not finding the association simply was a lack of statistical power. Finally, could you consider adding what is the meaning of these findings for public health or medicine?

**Abbreviations**

A list of all the abbreviations used would help the reader, including, for example, NFBC, CRHC, ICH, SAH, TIA, ICD, IS, BMI, etc.

**Table 1:**

Abbreviations used in tables should always be marked in legends.

Usually abbreviations are not used in headings (here ICD).

For a reader unfamiliar with ICD, it might not be clear with the first glance that these are codes. Maybe you could add the term “code” under ICD 8, 9 and 10 in the table.

**Table 2:**

Period is missing from the end of the heading.

Number of missing data (n) under “All” and the trajectory classes could be reported as well.

Terms used for education are not the same as the ones used in the methods.

Could “kg/m^2”^ and “g/day” be in parentheses?

**Table 3:**

Could “(including IS and TIA)” be marked under “Ischemic stroke” and “(including SAH and ICH)” under “Haemorrhagic stroke”?

Remember to add in legends the abbreviations used.

You should also state in legends with what variables were the models adjusted for and what does the text in bold mean.

Why the number of strokes before 31 years, between 31-46 and before 46 years are not applicable?

Use same words with ages in cumulative smoking (not by and before). Also, mark the unit which is years.

It is slightly difficult to understand from the Table 3 how to interpret the HR for cumulative smoking exposure. The text helps to understand it. Would there be a way to make this clearer also in the table?

**Figure 1:**

Please mark units for x-axis and y-axis.

Please mark sample sizes or proportions of participants in each trajectory group.

If possible, change the group numbers (1-5) to names of the groups within the figure.

**Supplement material 1:**

Why there is no information about the % of missing data from variable “use of other drugs at age 14” onward?

Apparently 97.2 % had missing data on “age of stroke onset”. Should this variable even be used?

**Supplement material 2:**

Missing all together. Needs to be added.
